# Supplementary material for: Differences between Rural and Urban Practices in the Response to the COVID-19 Pandemic: Outcomes from the PRICOV-19 Study in 38 Countries
Source: Int J Environ Res Public Health. 2023 Feb 19;20(4):3674. doi: 10.3390/ijerph20043674 (PMC9958860; doi:10.3390/ijerph20043674)
Supplement: Supplementary file 1 [file ijerph-20-03674-s001.zip › ijerph-2191576-supplementary.pdf]

Supplementary material

Supplementary Table S1: an overview survey questions and their original answer options that were the basis for the outcome variables

Survey question	Original answer options
Collaboration with other practices and experienced support	
If staff members in this practice are absent because of COVID-19 (infection or quarantine), this practice can count on the help of other PC practices in the neighborhoods	◎ Strongly disagree<br>◎ Disagree<br>◎ Neutral<br>◎ Agree<br>◎ Strongly agree<br>◎ I do not know
The COVID-19 pandemic has promoted cooperation with other PC practices in the neighborhoods	◎ Strongly disagree<br>◎ Disagree<br>◎ Neutral<br>◎ Agree<br>◎ Strongly agree<br>◎ I do not know
Since the COVID-19 pandemic, did you experience any limitations related to the building or the infrastructure of this practice to provide high-quality and safe care?	◎ To a large extent<br>◎ To a limited extent<br>◎ Hardly<br>◎ None<br>◎ I do not know
If staff members in this practice are absent because of COVID-19 (infection or quarantine), the work can be distributed in such a way that the well-being of colleagues is not compromised	◎ Strongly disagree<br>◎ Disagree<br>◎ Neutral<br>◎ Agree<br>◎ Strongly agree<br>◎ I do not know
The involvement of non-GP staff	
In the situation where telephonic triage is performed by someone other than a GP in this practice and he/she needs support when assessing a call, he/she can rely on support from a GP.	◎ Never<br>◎ Rarely<br>◎ Sometimes<br>◎ Usually<br>◎ Always<br>◎ I do not know<br>◎ Not applicable
Staff members are more involved in giving information and recommendations to patients contacting the practice by phone.	◎ Strongly disagree<br>◎ Disagree<br>◎ Neutral<br>◎ Agree<br>◎ Strongly agree<br>◎ I do not know/ not applicable
Patient safety incidents during COVID-19	
Due to the complexity of primary care and the high degree of uncertainty, incidents can occur in all practices. Please indicate whether the following incidents occurred in this practice since the COVID-19 pandemic:	
A patient with an urgent condition was seen late because he/she did not come to the practice sooner.	◎ Yes<br>◎ No<br>◎ I do not know
A patient with a serious condition was seen late because he/she did not know how to call on a GP.	◎ Yes<br>◎ No<br>◎ I do not know

A patient with an urgent condition was seen late, because the situation was assessed as non-urgent during the telephone triage.	◎ Yes<br>◎ No<br>◎ I do not know
A patient with a fever caused by an infection other than COVID-19 was seen late due to the fact the COVID-19 protocol was followed which delayed the care.	◎ Yes<br>◎ No<br>◎ I do not know
A list was compiled from the electronic medical record for at least one group of patients with a chronic disorder (e.g. all patients taking methotrexate and needing to be seen).	◎ Yes<br>◎ No<br>◎ I do not know
The use of protocols	
Is a protocol been used in this practice when answering phone calls from potential COVID-19 patients?	◎ Yes, this protocol is based on a government guideline<br>◎ Yes, this protocol is not based on a government guideline<br>◎ No<br>◎ I do not know
In this practice, sufficient time is provided between consultations for the disinfection of the consultation room.	◎ Never<br>◎ Rarely<br>◎ Sometimes<br>◎ Usually<br>◎ Always<br>◎ I do not know/not applicable
Information for patients	
Does the answering machine of this practice provide information in multiple languages?	◎ Yes, in multiple languages<br>◎ No<br>◎ I do not know<br>◎ There is no answering machine
Is the leaflet of this practice available to patients in multiple languages?	◎ Yes, in multiple languages<br>◎ No<br>◎ I do not know<br>◎ There is no practice leaflet
Does this practice have a leaflet with information on COVID-19 to give to patients?	◎ Yes, in one language<br>◎ Yes, in multiple languages<br>◎ No<br>◎ I do not know
Is the information on the website of this practice available in multiple languages?	◎ Yes, in multiple languages<br>◎ No<br>◎ I do not know<br>◎ There is no website
Initiatives for vulnerable patients	
A list was compiled from the EMR (*) for at least one group of patients with a chronic disorder (e.g. all patients taking methotrexate and needing to be seen).	◎ Yes<br>◎ No<br>◎ I do not know
This practice contacted patients with a chronic condition who needed follow-up care.	◎ Yes<br>◎ No<br>◎ I do not know

This practice contacted psychologically vulnerable patients.	◎ Yes<br>◎ No<br>◎ I do not know
This practice contacted patients with previous problems of domestic violence or with a problematic child-rearing situation	◎ Yes<br>◎ No<br>◎ I do not know
When a patient needs to isolate him/herself, the extent to which this is feasible at his/her home is checked with the patient.	◎ Never<br>◎ Rarely<br>◎ Sometimes<br>◎ Usually<br>◎ Always<br>◎ I do not know

Since the pandemic the role of non-GP staff members might have changed. Please rate how much you agree with the following statement since the COVID-19 pandemic:

Since the COVID-19 pandemic, staff members are more involved in giving information or explaining what a caregiver has said to illiterate patients, patients with low health literacy or migrants.	◎ Strongly disagree<br>◎ Disagree<br>◎ Neutral<br>◎ Agree<br>◎ Strongly agree<br>◎ I do not know/ not applicable
Since the COVID-19 pandemic, staff members are more involved in actively reaching out to patients that might postpone healthcare.	◎ Strongly disagree<br>◎ Disagree<br>◎ Neutral<br>◎ Agree<br>◎ Strongly agree<br>◎ I do not know/ not applicable
